# Assessing the ecological risk of representative wastewater based on a growth inhibition method with freshwater algae (*Raphidocelis subcapitata*)

**DOI:** 10.1007/s44297-023-00007-8

**Published:** 2023-09-11

**Authors:** Aoxue Wang, Hanqing Li, Tao Liang, Gang Lian, Wenjun Gui, Shengli Zhou, Shuying Li

**Affiliations:** 1 Institute of Pesticide and Environmental Toxicology, College of Agriculture and Biotechnology, Zhejiang University, Hangzhou, 310058 People’s Republic of China; 2 Zhejiang Province Ecological Environment Monitoring Centre, Hangzhou, 310012 People’s Republic of China; 3 Zhejiang Key Laboratory of Ecological and Environmental Monitoring, Forewarning and Quality Control, Zhejiang University, Hangzhou, China; 4 Ministry of Agriculture Key Laboratory of Molecular Biology of Crop Pathogens and Insects, Zhejiang University, Hangzhou, China; 5 Zhejiang Provincial Key Lab of Biology of Crop Pathogens and Insects, Zhejiang University, Hangzhou, China

**Keywords:** *Raphidocelis subcapitata*, Microalgae, Water quality, Chlorophyll fluorescence, Toxicity, Environmental risk

## Abstract

Wastewater contains a vast number of pollutants that potentially threaten the ecosystem after being discharged into the environment. The present study aimed to develop a rapid and sensitive toxicity detection method for assessing the ecological risk of wastewater. *Raphidocelis subcapitata* was used as the test organism in the algal growth inhibition test, and the anti-interference capacity of *R. subcapitata* itself and three biomass measuring methods (spectrophotometer, electron particle counter and chlorophyll fluorescence) on sample color and sample particulate matter were examined. The results revealed that chlorophyll fluorometry was more suitable for determining biomass in the growth inhibition test. Furthermore, chlorophyll fluorometry was selected to evaluate the combined toxic effects and sensitivity of *R. subcapitata* to wastewater from different industries. Microalgae respond differently to the toxicity of wastewater from various sources. The LID values for chemical wastewater 2, urban sewage 1, pharmaceutical wastewater 1, electroplating effluent and pharmaceutical wastewater 2 were 4.0, 3.0, 2.7, 2.4 and 2.0, respectively. The present study investigated the toxicity response of *R. subcapitata* to wastewater from various industries by growth inhibition experiments to comprehend the possible ecological concerns of wastewater from diverse businesses. This study will provide a significant basis for the design of detection methods and a comprehensive evaluation of toxicity in surface water environments.

**Graphical Abstract:**

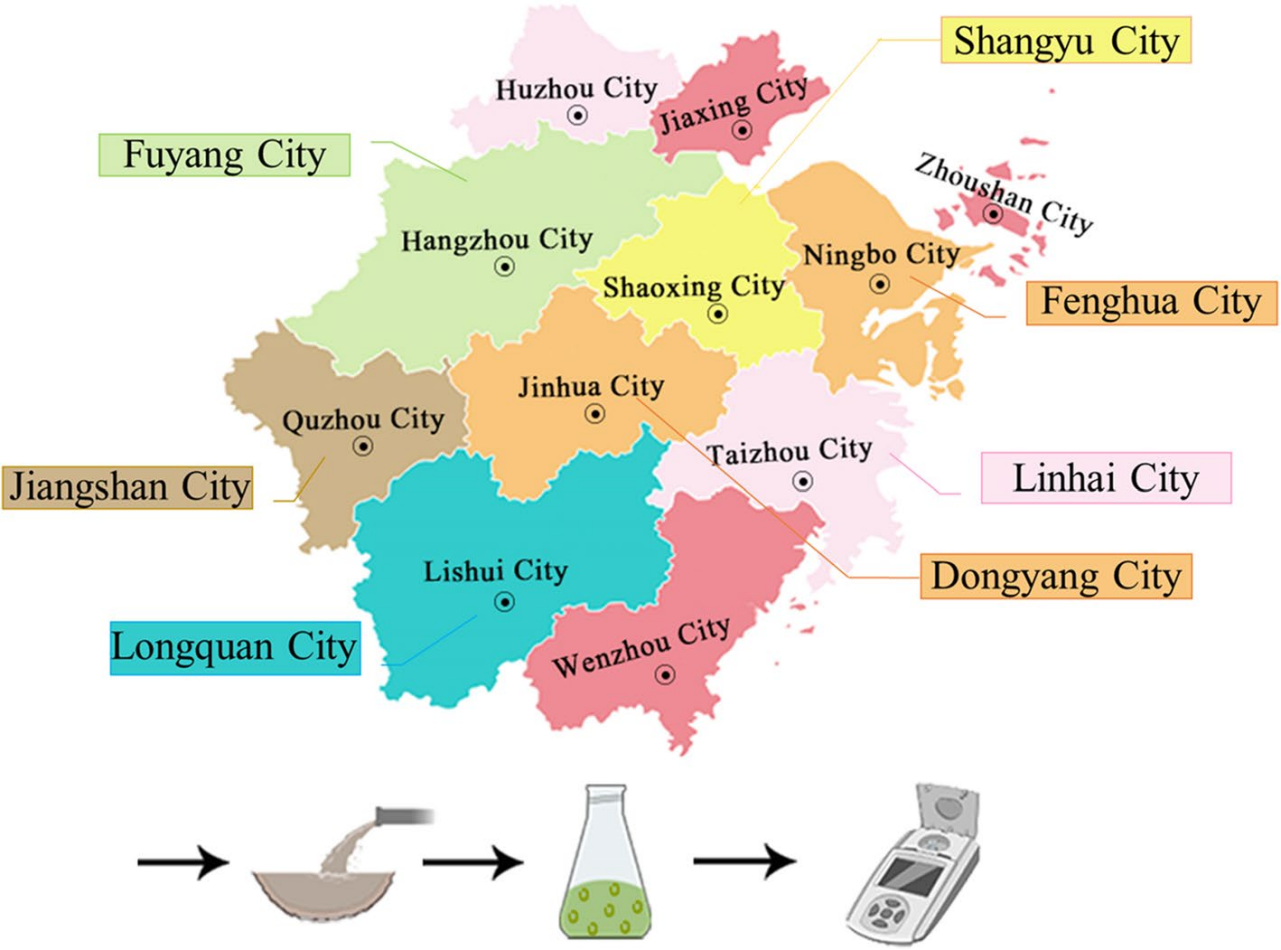

**Supplementary Information:**

The online version contains supplementary material available at 10.1007/s44297-023-00007-8.

## Introduction

The comprehensive discharge of industrial and agricultural wastewater and household sewage has continuously risen in recent years, resulting from the ongoing expansion of industry and agriculture as well as the acceleration of urbanization. Both technologically advanced and developing countries face increasingly serious environmental problems caused by the release of toxic pollutants into the environment. Many studies have demonstrated that the sewage treatment process falls short in its removal of poisonous and dangerous compounds, as evidenced by the fact that after treatment, persistent organic pollutants (POPs), disinfection byproducts, plasticizers, antibiotics, and pesticides are frequently still detected [1–6].

The water quality is reduced by wastewater with increasing salinity, total dissolved solids (TDS), total suspended solids (TSS), biological oxygen demand (BOD) and chemical oxygen demand (COD) [7]. Many biological studies have demonstrated the potential hazard of urban sewage and various industrial drainages to cause a variety of acute and chronic toxicities at the cellular and individual levels, including endocrine disrupting toxicity, genetic toxicity, developmental toxicity, etc. [8–11]. Without proper management, it will gravely endanger human health and the security of the aquatic ecosystem once different types of wastewater have been released into the environment. For example, the findings of pertinent studies conducted at home and abroad have demonstrated that, despite varying degrees of dilution, some natural waters still exhibit chronic toxic effects, such as endocrine disruption, genetic and developmental effects, and even acute toxic effects, including activity inhibition and lethal effects [12–14]. Therefore, it is necessary to establish quality standards for industrial or municipal wastewater discharges to protect the aquatic environment and achieve sustainable management. Although it is widespread practice, chemical analysis of wastewater does not account for hazardous effects on organisms. Thus, it is crucial to evaluate sewage quality biologically [7].

Many regulatory agencies assess the environmental toxicity of discharges to surface waters by using aquatic invertebrates (e.g., *Ceriodaphnia dubia*, *Daphnia magna*), fish (e.g., *Pimephales promelas*), bacteria (e.g., *Pseudomonas putida*), microalgae (e.g., *Pseudokirchneriella subcapitata*) and higher plants (e.g., *Lemna minor*) in a series of experiments by Halleux et al. [15]. However, for aquatic bioassays, algae seem to be the more appropriate organisms with a higher sensitivity.

Microalgae are an essential component of the marine nutrient chain and a major producer in the aquatic environment. They play a crucial role in preserving the balance of the marine environment and are necessary for the appropriate structure and operation of the overall ecosystem [16, 17]. Higher nutrition levels may be impacted by production level disruptions [18]. Microalgae also benefit from a quick growth cycle, easy maintenance, simple monitoring, and sensitivity to toxins [19]. Planktonic algae are further sensitive indicators for evaluating the various consequences of contaminant release into the ocean. Algae are more vulnerable to various water contaminants and industrial effluents than invertebrates and fish, according to certain research [20–23].

*Raphidocelis subcapitata* (formerly known as *Pseudokirchneriella subcapitata and Selenastrum capricornutum*, UTEX 1648) is one of the most sensitive algal species [24]. Various environmental samples, such as wastewater samples, leachate, surface water and soil elutriates, as well as chemicals and mixtures, are ecotoxicologically characterized using algal growth inhibition tests. Biological toxicity tests developed by the US Environmental Protection Agency (US E.P.A.) and the Organization for Economic Co-operation and Development (OECD) all set *R. subcapitata* as the test organism.

Photosynthesis is the most basic physiological process of microalgae. Most of the light energy absorbed by chlorophyll is used for photosynthesis, and the part that cannot be utilized will be emitted in the form of heat and fluorescence. Due to the mutual competition among these three methods for energy, the change in photosynthesis can cause the corresponding change in fluorescence emission [25]. When contaminants interact with algal cells, the toxic effects of contaminants on algae can be expressed through photosynthesis and then cause a change in chlorophyll fluorescence. According to Muller et al. [26], chlorophyll a fluorescence analysis is a bioanalytical approach that may be used to assess exposure to combinations of contaminants that behave in a similar manner. Chlorophyll a fluorescence measurements were recommended as a useful method for assessing the toxicity of herbicides on aquatic plants and algae [27]. A potent technique for studying the ecophysiology of phytoplankton and tracking its biomass is chlorophyll a fluorescence [26]. Therefore, a novel method for the thorough evaluation of water pollution has emerged: employing chlorophyll fluorescence as a probe for biological toxicity studies.

The aim of this study was to test and optimize a miniaturized and low-cost algal growth-inhibition assay based on the ISO standard [28]. Chlorophyll fluorescence and the algae *R. subcapitata* were used as tools for pollutant phytotoxicity screening. In this study, three approaches (spectrophotometer, electron particle counter and chlorophyll fluorescence) were used to evaluate the performance of the miniaturized algal growth-inhibition assay with *R. subcapitata.* We compared their anti-interference capability to sample color and particle matter, as well as their association with algal density. Finally, chlorophyll fluorescence was selected to validate the algal growth inhibition test on seven actual water samples, including two urban wastewater samples, two chemical wastewater samples, two pharmaceutical wastewater samples and one electroplating wastewater sample. Our results provide an important basis for the development of a rapid and sensitive detection method for water sample ecological risk assessment.

## Materials and methods

### Sample collection and pretreatment

**Fig. 1:**
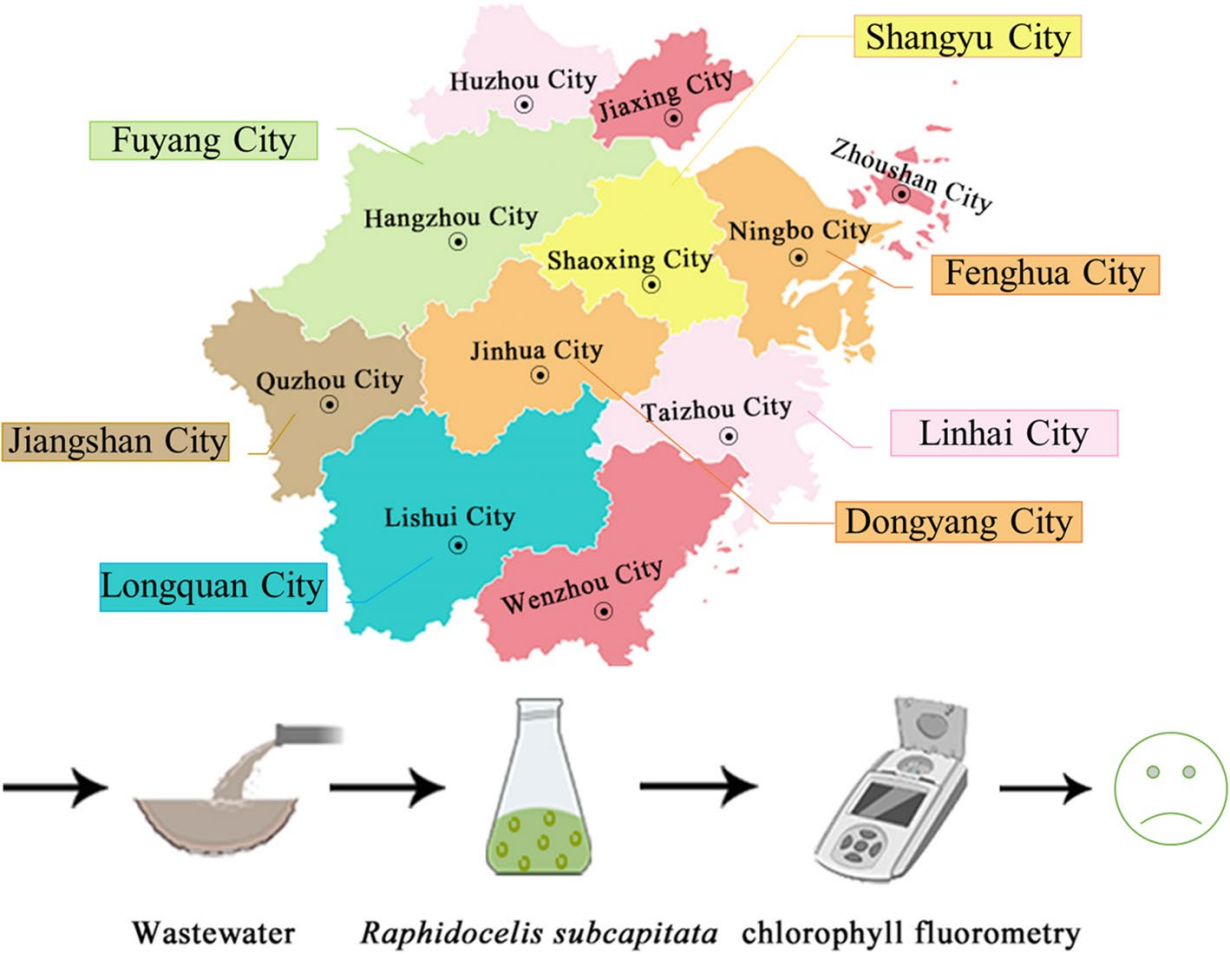
Seven wastewater sample points in Zhejiang Province, China

Seven wastewater samples were collected from various parts of Zhejiang Province (Fig. 1): urban sewage 1 (Us1), urban sewage 2 (Us2), chemical wastewater 1 (Cw1), chemical wastewater 2 (Cw2), pharmaceutical wastewater 1 (Pw1), pharmaceutical wastewater 2 (Pw2), and electroplating effluent (Ee) were tested with the algal test (chlorophyll fluorescence). Each sample was collected in polystyrene containers, divided into aliquots (100 and 250 mL) and stored at -20°C for 2 months.

Environmental samples were thawed at no higher than 25 °C and used immediately prior to the toxicity test. To precipitate particulate matter from the samples, the thawed samples were centrifuged at 4,500 × g for 10 min. The supernatant was finally taken for the test. Growth inhibition tests were performed on the above seven actual water samples at 6 h, 48 h and 2 months after sampling to verify the shelf life of the water samples.

For the lowest ineffective dilution (LID) of wastewater to be determined, the following dilution series was used: 1:1, 1:2, 1:3, 1:4, 1:6, 1:8, and 1:12, and the dilution series of tested chemical compounds was prepared using ISO medium [28]. Nutrient salt stock solution equal to the control growth medium was added to the undiluted samples before performing dilution of the water samples (Supplementary Information, SI Table S1).

### Test algae and preculture

The algal species *R. subcapitata* was obtained from the Freshwater Algae Culture Collection at the Institute of Hydrobiology, Wuhan, China, and cultured in the laboratory following OECD guidelines No. 201 [29]. The algae were maintained and precultured in ISO medium (ISO 8692) [28] under continuous cool white light luminescent tubes (6,000–10,000 lx) in axenic conditions and continuously shaken (100–200 rpm) at 23 ± 2°C. The cell density of the preculture prepared algal solution was kept ^between 5×105^ cells/mL and 10^6^ cells/mL and then used for the subsequent experiment. Generally, 3 days of algal culture was used for the inoculation of tested concentrations and controls.

### Method optimization

As light is the primary source of energy for algae development, variations in light intensity may have an impact on the growth rate. By obstructing or filtering out specific wavelengths of light necessary for algae growth, water color and particle matter can have an impact on algae growth. Because of the decreased growth rate brought on by shadowing, the alga may become less susceptible to harmful effects, hiding any chemical toxicity [30]. As a result, the color and particle content of water samples will be significant hindrances to algae development. The most significant interferences in the algal growth inhibition test occur during the algal growth culture procedure and the growth measurement, which is directly connected to the measurement technique.

#### Relationship between different biomass determination methods and algal cell density

Cell counting is the basic alternative method for measuring biomass in algal growth inhibition tests. They can be counted by microscopes and electron particle counters [28, 29], and other alternative parameters, such as chlorophyll fluorescence and spectrophotometry, can also be used to measure biomass. However, there are obvious differences in the regulations on the optical diameter length of the absorption pool of the spectrophotometer.

By using an electron particle counter and scanning the absorption spectra, the particle size distribution, fluorescence properties, and absorption peak of *R. subcapitata* were determined in this study. Additionally, the algal cell density was determined by microscopic counting of high-concentration algal fluid, and an algal fluid diluent with 5 cell density gradients (ranging from 3.2 × 10^3^—1 × 10^6^ cells/mL) was prepared in accordance with the initial inoculation concentration and growth multiple of the *R. subcapitata* growth inhibition test. The measurement was performed using an electron particle counter with a 2–14 μm particle size range, a multichannel chlorophyll fluorescence spectrometer with excitation wavelengths of 470, 520, 645, and 665 nm, absorption wavelengths of 682 nm, and a 5 cm absorption cell spectrophotometer.

#### Disturbance and elimination of growth

To simulate various sample color depths, 5 different concentrations of 1, 3.2, 10, 32, and 100 mg/L reactive brilliant blue KN-R solid were prepared using medium. In the ball mill, the ceramic rings were totally crushed to powder. The suspension was then created, and it was allowed to set for 24 h. The higher turbidity solution was further diluted with the medium into five turbidity ratios of 25, 50, 100, 200, and 400 porcelain powder concentrations (NTU) to simulate samples with varied particle concentrations. To test the anti-interference ability of *R. subcapitata* on sample color and sample particles. The growth inhibition tests were carried out under the conditions of 6000, 8000 and 10000 lx light intensity, and the algal density was 1 × 10^4^ cells/mL.

#### Measurement of interference and elimination

In terms of sample color and particulate matter interference, the spectrophotometer, electronic particle counter, and chlorophyll fluorescence were compared. To replicate the color and particle matter of the materials as mentioned in the previous approach, porcelain rings and activated brilliant blue were utilized. To rectify the results, identical concentrations of porcelain powder and activated brilliant blue were calculated without algae, and the accuracy and precision of the corrected measurements were confirmed and assessed.

### Validity of the test

The number of algae cells in the control wells must increase by at least a factor of 16 during the 72 h test period for the toxicity test to be acceptable. The test was performed with potassium dichromate and 3,5-dichlorophenol as reference chemicals. The concentrations of 3,5-dichlorophenol were set at 4.00, 3.48, 3.02, 2.63, 2.29, 1.99 and 1.73 mg/L, the concentrations of potassium dichromate were 1.44, 1.20, 1.00, 0.83, 0.69, and 0.58 mg/L, and a total of six tests were conducted.

### Algal growth inhibition test

The algal inoculum for the test was taken from an exponentially growing preculture of *R. subcapitata*, which was set up 3 or 4 days before the start of the test. After the diluted water sample was brought to a constant temperature of 23 ± 2°C away from light, algal medium without samples was used as a blank control. The precultured algal solution was inoculated into the medium and the diluted water sample at an inoculum volume of 1:100 (e.g., 100 mL of diluted water sample was inoculated with 1 mL of algal solution) to prepare the test solution. The initial density of algal cells in the test solution was 5 × 10^3^ cells/mL—10^4^ cells/mL.

In each test vessel, the original algal cell density and test solution volume were kept constant. For each diluted concentration water sample, three parallels were set up, and six parallels were set up for the blank control. The solvent DMSO was used at a final concentration of 0.02% v/v at all concentrations of the tested compounds and in control cultures. This concentration has been previously demonstrated to have no effect on the growth of *R. subcapitata* [31]. The test vessel was incubated at 23 ± 2°C under continuous white light while being shaken continuously at 100–200 rpm. During the exposure every 24 h, the cell morphology was checked by light microscopy, and growth was determined by removing 70 µL fractions from each test sample for cell counting by flow cytometry [32]. After 72 h, algal cells were counted using chlorophyll fluorescence. Growth rates were calculated according to the OECD 201 guidelines, and the results were expressed as growth rate inhibition relative to the untreated control, and LID was obtained. Test validity was confirmed by the following control criteria: the average specific growth rate of the control group was at least 1.4 d^−1^, the coefficient of variation of each parallel ratio growth rate in the control group should be < 5%, and the pH value of the control group should not change by more than 1.5 units [28].

### Data analysis

One-way analysis of variance (ANOVA) was performed on the data using SPSS software, and *p* < 0.05 was considered statistically significant according to Tukey's test. The specific growth rate for each parallel of the control and diluted water samples was calculated according to equation [1]:

1 $$\mu = \frac{1n\,{n}_{L}-1n\,{n}_{0}}{{t}_{L}- {t}_{0}}$$

where:

*μ* is the average specific growth rate, d^-1^;

*n*_*0*_ is the initial algal cell density;

*n*_*L*_ is the algal cell density measured at t_L;_

*t*_*0*_ is the test start time, d;

*t*_*L*_ is the time of the end of the experiment or the last measurement within the growth period of the control index, d

The growth inhibition rate based on the specific growth rate for each parallel of the diluted water samples was calculated according to equation [2]:

2 $${I}_{\mu i}= \frac{{\mu }_{C}- {\mu }_{i}}{{\mu }_{C}} \times 100$$

where:

*I* ${\mu }_{i}$ is the growth inhibition rate, %;

*μ*_*C*_ is the mean value of each parallel ratio growth rate in the control group, d^−1^;

*μ*_*i*_ is the specific growth rate of each parallel of the water sample, d^−1^

## Results

### Validation of sample shelf life

The results showed that the samples remained valid within the above storage methods and storage times (SI Table S2), and the standard deviation of LID for all samples at different storage times was less than or equal to the standard deviation of 6 independent tests.

### Method establishment and optimization

#### Algal cell density and determination methods

The electron particle counter showed that the particle size of *R. subcapitata*. was mainly between 2.5 μm and 7 μm, and the peak value was approximately 4.5 μm. The absorption spectra of *R. subcapitata* were scanned at 340–700 nm, and there were 5 absorption peaks at 364 nm, 384 nm, 438 nm, 624 nm and 682 nm. The fluorescence characteristics of *R. subcapitata* were also determined (SI Figure S1). Good linear relations were found by using the three methods. The R2 value of the electron particle counting method was 0.9996 and that of the chlorophyll fluorescence and spectrophotometry method was 0.9997 (SI Figure S2). The parallel coefficients of variation of the above methods ranged from 0.65% to 5.14%, 0.70% to 3.07% and 0.73% to 13.32%, respectively (SI Table S3). For the determination of high algal density, there was no significant difference in the accuracy of the methods, and the coefficient of variation was less than 5%. However, for the determination of algal densities of 3.2 × 10^3^ cells/mL and 1 × 10^4^ cells/mL, the accuracy of the spectrophotometric method was significantly lower than that of chlorophyll fluorescence and electron particle counting.

#### Growth disruption and elimination

**Fig. 2:**
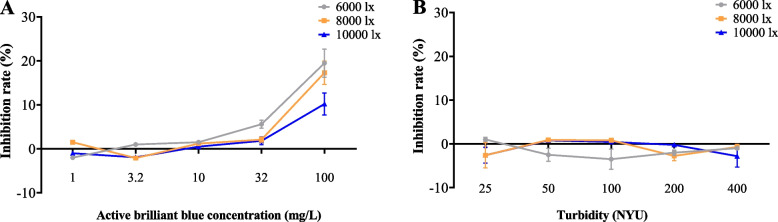
Antidisturbance of sample color and sample particulate matter by *R. subcapitata*. **A** The abscissa is the concentration of active brilliant blue. **B** The abscissa is the turbidity. The growth inhibition rate (%) is represented on both ordinates (compared with the control). Error bars represent standard deviation

At 6000, 8000, and 10000 lx, the results showed potent anti-interference abilities of *R. subcapitata*, and growth inhibition only occurred when the active brilliant blue concentration was over 10 mg/L and was inversely correlated with light intensity (Fig. 2A). The growth of *R. subcapitata* exhibited a good anti-interference capacity for the particulate matter in the sample, according to the results of the particulate matter of the water samples interfering with it. When the turbidity was 400 NTU and below, the particles in the samples exhibited no appreciable growth inhibiting impact on *R. subcapitata* compared to the blank control (Fig. 2B).

#### Influence of sample color on the determination method

**Fig. 3:**
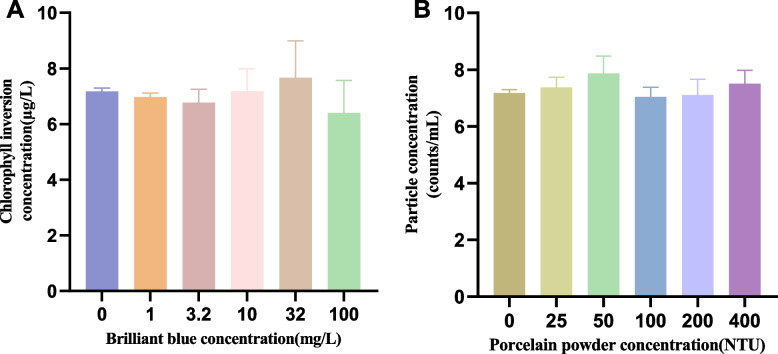
Interference of sample color and particulate matter in the determination of chlorophyll fluorescence (mean ± S.D., *N* = 3). **A** The abscissa is the concentration of active brilliant blue, and the ordinate is the chlorophyll inversion concentration. **B** The abscissa is the concentration of PP, and the ordinate is the particle concentration. Error bars represent standard deviation

Expression of sample color interference showed that using chlorophyll fluorescence (Fig. 3A), the interference of color had no discernible impact on the corrected measurements when the active brilliant blue concentration was less than 3.2 mg/L. The coefficient of variation (RE) between the measured results and the reference value (when the concentration of active brilliant blue was 0) and the relative standard deviation (RSD) of the measured data were both less than ± 10%. However, as the concentrations of active brilliant blue increased, both RSD and RE progressively increased (SI Table S4). The color interference of the sample had no discernible impact on the corrected measurement results when the concentration of active brilliant blue was 3.2 mg/L and below, according to the verification results of the color sample interference spectrophotometer (SI Table S5). However, as the color of the sample darkened, the value of RE increased sharply.

#### Effect of sample particulate matter on the determination method

Data from the chlorophyll fluorescence analyzer showed that no significant impacts were found from different particulate matter concentrations (Fig. 3B). The values of RSD and RE were less than ± 10% (SI Table S6). According to the verification results of the electronic particle counter (SI Table S7), the interference from particulate matter had no appreciable impact on the measurement outcomes when the turbidity was less than 100 NTU. However, the values of RSD and RE increase sharply with increasing particulate matter concentrations. Different concentrations of sample particulate matter have a significant influence on the determinations by using the spectrophotometer (SI Table S8). The values of RSD and RE were too large.

### Reference substance verification

**Fig. 4:**
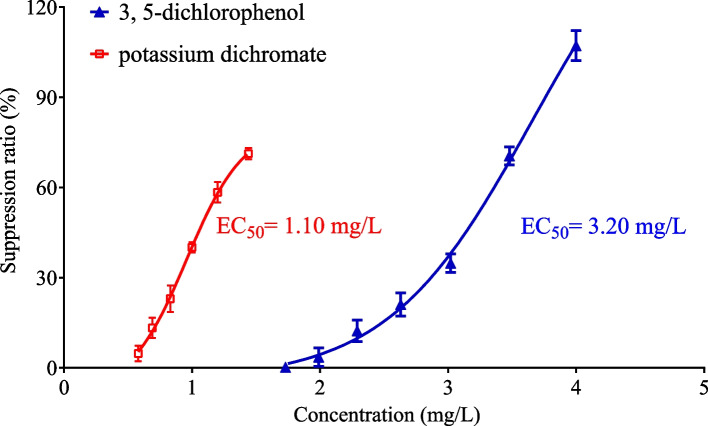
Suppression ratio of *R. subcapitata* by potassium dichromate and 3,5-dichlorophenol using the optimized method. Data are the results of 6 independent tests

Algal growth inhibition tests with the reference substances 3,5-dichlorophenol and potassium dichromate were conducted (SI Table S9). The average EC_50_ of the potassium dichromate test during the experiment was 1.10 mg/L, and the average EC_50_ of the 3,5-dichlorophenol test was 3.20 mg/L (Fig. 4). The verification results indicated that the proposed method is suitable for the risk assessment of algal growth inhibition by hazardous substances.

### Environmental water sample assay

The results of six independent laboratory tests on effluent samples revealed that the LID values of Cw2 and Us1 were relatively similar to those of *R. subcapitata* at 72 h, while Pw2 had the lowest LID at that time. The LID for wastewater samples from urban sewage 2 (Us2) and chemical wastewater 1 (Cw1) was 7.0. This indicated that *R. subcapitata* is most sensitive to effluent samples from Us2 and Cw1. As shown in Table 1, Cw1 and Ee at low concentrations had a greater effect on the growth inhibition of *R. subcapitata*. With the dilution of the water sample concentration, the growth inhibition rate of *R. subcapitata* at 72 h gradually weakened. Pw2 had less effect on the growth of *R. subcapitata* (from -2.8% to 25.4% at 72 h).

**Table 1:** Results of the algal growth inhibition test on environmental water samples

Sample^a^	Test number^b^	Dilution ratio	Suppression ratio		
			$\overline X$	Si	CV
Us1	6	1	47.8%	3.7%	7.7%
		2	8.7%	2.2%	25.4%
		3	0.0%	1.1%	3038.0%
		LID	3.0	0	0
Us2	6	1	65.9%	4.4%	6.6%
		2	44.8%	4.0%	9.0%
		3	17.1%	3.1%	18.0%
		4	11.0%	2.2%	20.4%
		6	5.1%	2.1%	41.4%
		8	2.1%	1.5%	73.6%
		12	-0.8%	1.5%	-193.2%
		LID	7.0	1.1	15.6%
Cw1	6	1	97.4%	4.4%	4.6%
		2	62.4%	5.1%	8.2%
		3	24.6%	7.1%	29.0%
		4	13.2%	4.1%	31.2%
		6	3.9%	2.2%	55.9%
		8	-1.0%	1.4%	-132.2%
		12	-1.2%	0.6%	-49.1%
		LID	7.0	1.1	15.6%
Cw2	6	1	57.8%	1.4%	2.4%
		2	23.6%	2.3%	9.8%
		3	6.9%	1.3%	19.0%
		4	1.2%	0.6%	51.5%
		LID	4.0	0	0
Pw1	6	1	39.2%	3.6%	9.2%
		2	5.0%	2.0%	41.1%
		3	0.1%	2.1%	1678.3%
		LID	2.7	0.5	19.4%
Pw2	6	1	25.4%	2.0%	8.0%
		2	-2.3%	0.9%	-39.1%
		3	-2.8%	1.6%	-56.7%
		LID	2.0	0	0
Ee	6	1	91.8%	6.1%	6.6%
		2	13.8%	3.0%	21.6%
		3	1.1%	0.7%	64.0%
		LID	2.4	0	0

^a^Wastewater samples from several locations: *Us* Urban sewage, *Cw* Chemical wastewater, *Pw* Pharmaceutical wastewater, *Ee* Electroplating effluent

^b^Data are the results of 6 independent tests. $\overline X$  is the average value, Si is the standard deviation, and CV is the coefficient of variation

## Discussion

In this work, a miniaturized and low-cost algal growth inhibition assay including *R. subcapitata* was tested and optimized according to the ISO 8692 standard. Potassium dichromate and 3,5-dichlorophenol were used as positive references, and good results were obtained. We focused on the growth inhibition of algae, which are representatives of primary producers and play a key role in aquatic ecosystems. It is also the basis of the aquatic food chain; therefore, the death of algae will affect aquatic organisms at higher trophic levels (e.g., secondary poisoning and food reduction) [33]. Previous comparative studies on algal toxicity testing using different algal species or different assay methodologies have been carried out by several studies [18, 34, 35]. According to a sensitivity rank of seven algal species, *Raphidocelis subcapitata* (syn. *P. subcapitata*) was assigned as the most sensitive species [24]. *Raphidocelis subcapitata* is widely used worldwide for bioassays in toxicological risk assessments [36]. Algal bioassays have been successfully applied to evaluate the impact of phytotoxic compounds in environmental samples. A simple algal bioassay guided by chemical analysis is an effective tool to better understand the ecological impact of agricultural discharges on receiving water bodies [37]. For example, in a static 14-day test, treated aluminum plating factory effluents showed a modest level of toxicity to *D. tertiolecta*. *D. tertiolecta* were stimulated to 40% (v/v) with the untreated and treated effluents from the pharmaceutical factory and were inhibited at greater concentrations. Published results indicated that although the treated wastewater met the discharge standards, these standards cannot fully protect the aquatic environment [38]. Gan et al. employed the marine diatom *Nitzschia closterium* as a test organism to select the best chlorophyll fluorescence parameters for a rapid and sensitive determination of lead on *N. closterium* based on the chlorophyll fluorescence technique [25]. Wang et al. revealed that PSNPs-SO_3_H contributed to more severe growth inhibition of *Microcystis aeruginosa* than PSNPs [39]. Kusk et al. determined the toxicity of 425 organic chemical substances to *Pseudokirchneriella subcapitata* using growth inhibition tests of algae, of which 94 substances had EC_50_ values below 1 mg/L and should be classified as very toxic [40]. Thus, algal bioassays have been successfully applied to evaluate the impact of phytotoxic compounds in environmental samples.

When the sensitivity of the method is adequate and the results are highly correlated with algal cell density, other measurements can be used instead of cell density, such as using electronic particle counters, chlorophyll fluorescence, and spectrophotometers. Cell counting is a fundamental alternative method for biomass determination in algal growth inhibition assays and can be carried out using microscopy and electronic particle counters. In this study, three different approaches to determining biomass were compared, including electronic particle counting, spectrophotometry, and chlorophyll fluorescence. The results revealed that chlorophyll fluorescence and spectrophotometry have some anti-interference ability to the color of the sample when the background color of the sample and the contribution ratio of algae to the measured value are less than 2.7 and 1.4, respectively. The anti-interference ability of the sample particles from high to low is chlorophyll fluorescence, electron particle counter and spectrophotometer. The accuracy of the chlorophyll fluorescence could meet the test requirements at all particulate matter concentrations by measuring equal concentrations of samples without algae to correct the measurement results. Chlorophyll concentration is a reliable predictor of algal biomass, and OECD TG 201 and other ISO recommendations for the algal growth test propose both in vivo and in vitro fluorescence measurements [41]. In conclusion, chlorophyll fluorescence is a reliable and rapid way to calculate the density of algal cells.

Industrial facility effluent discharges are intricate, containing a wide range of components and fluctuating constantly in both amount and quality. In this study, the *R. subcapitata* growth inhibition test was used to evaluate seven wastewaters from various sectors. Us2 and Cw1 had the highest LID values with LID = 7.0, and all seven of the actual water samples analyzed had LID values that were within the discharge limits for algal growth inhibition specified in China's *Discharge Standards for Pollutants from Urban Wastewater Treatment Plants* [42]. In some cases, the treated effluent did not exceed the discharge limits, but the results of toxicity tests showed potential toxicity [43]. In fact, some chemicals were not eliminated, as the conventional technology of treatment used in wastewater treatment plants appears to be insufficient for completely removing these specific compounds [44]. Medications could have hazardous synergistic effects when there were other substances present in the effluents, according to Cleuvers [45]. Although no hazard to fish (*Lepistes sp.*) was found, treated pharmaceutical factory plant effluent from the analgesic and anti-inflammatory medication production line was stimulatory to the investigated algae at low concentrations and inhibitory at high concentrations [38]. In our investigation, *R. subcapitata* was inhibited by two different types of pharmaceutical wastewater at dilution 1 at rates of 39.2% and 25.4%. However, as the dilution ratio of the water sample increased, the growth inhibition rates of Pw2 on *R. subcapitata* were -2.3% and -2.8%, suggesting that they may have negative effects on the aquatic environment by promoting algal growth at low concentrations. Reduced water clarity and increased oxygen consumption in bottom waters after settling are the two main effects of algal overproductivity [38]. Water quality may be significantly influenced if the biomass of algae increases too much or if specific species become prevalent. Lower water clarity may affect fish population shifts and the ability of higher-order vascular aquatic plants to develop and survive. Furthermore, the long-term effects of continued low-level exposure to chemicals and their metabolites are unclear. Therefore, comprehensive water quality toxicity monitoring and water ecological health risk assessment using algal growth inhibition tests are necessary.

## Conclusion

The chlorophyll fluorescence method was developed in this study to examine the cumulative toxic effects of several wastewater samples on the growth inhibition of *R. subcapitata*. To define the harmful effects of the tested water samples on the suppression of the development of the algae, the findings were assessed in terms of the lowest ineffective dilution (LID). The results demonstrated that the combined toxicity of the seven actual water samples measured within the discharge limits for algal growth inhibition specified in China's *Discharge Standards for Pollutants from Urban Wastewater Treatment Plants *[42]. The approach utilized in this study is also adaptable to surface water, groundwater, household wastewater, and industrial wastewater. However, ecological risks need to be further assessed, as a growth inhibition rate on *R. subcapitata* was found. Overall, this method meets the requirements of relevant environmental management standards and environmental protection work and has strong feasibility and operability.

## Supplementary Information

**Additional file 1.**

## Data Availability

Not applicable.
