# Supplementary material for: Assessing the ecological risk of representative wastewater based on a growth inhibition method with freshwater algae (Raphidocelis subcapitata)
Source: Crop Health. 2023 Sep 11;1(1):7. doi: 10.1007/s44297-023-00007-8 (PMC12825988; doi:10.1007/s44297-023-00007-8)
Supplement: Supplementary file 1 — Additional file 1. [file 44297_2023_7_MOESM1_ESM.docx]

**Supplementary Information**

Assessing ecological risk of representative wastewater based on a growth inhibition method with freshwater algae (*Raphidocelis subcapitata*)

Aoxue Wang ^a^, Hanqing Li ^a^, Tao Liang ^b,e^, Gang Lian ^b,e^, Wenjun Gui ^a,c,d^, Shengli Zhou ^b,e*^ Shuying Li ^a,c,d,*^

^a^ Institute of Pesticide and Environmental Toxicology, College of Agriculture and Biotechnology, Zhejiang University, Hangzhou, 310058, PR China

^b^ Zhejiang Province Ecological Environment Monitoring Centre, Hangzhou, 310012, PR China

^c^ Ministry of Agriculture Key Laboratory of Molecular Biology of Crop Pathogens and Insects, Zhejiang University, Hangzhou, China

^d^ Zhejiang Provincial Key Lab of Biology of Crop Pathogens and Insects, Zhejiang University, Hangzhou, China

^e^ Zhejiang Key Laboratory of Ecological and Environmental Monitoring, Forewarning and Quality Control, Zhejiang University, Hangzhou, China

*Corresponding Author:

**Shuying Li** - Institute of Pesticide and Environmental Toxicology, Zhejiang University, Hangzhou, 310058, PR China. E-mail: [lishuyingabc@zju.edu.cn](mailto:lishuyingabc@zju.edu.cn)

**Shengli Zhou** - Zhejiang Province Ecological Environment Monitoring Centre, Hangzhou, 310012, PR China. E-mail: slzhou2002@163.com

Table S1. Dilution of water samples

| Dilution ratio D | Total integral number of the original water sample in the diluted water sample (%) | Water sample after pretreatment | Composition of Diluted Water Sample (mL) | | |
| --- | --- | --- | --- | --- | --- |
|  |  |  | Required culture-medium | Volume of water sample required | Volume of water after diluting (mL) |
| 1 | 100.0 | Dilute water sample 1 time | 0 | Water sample after pretreatment 800 | 800 |
| 2 | 50.0 | Dilute water sample 2 time | 400 | Water sample after pretreatment 400 | 800 |
| 3 | 33.3 | Dilute water sample 3 time | 540 | Water sample after pretreatment 270 | 810 |
| 4 | 25.0 | Dilute water sample 4 time | 400 | Dilute water sample twice 400 | 800 |
| 6 | 16.7 | Dilute water sample 6 time | 400 | Dilute the water sample 3 times 400 | 800 |
| 8 | 12.5 | Dilute water sample 8 time | 400 | Dilute the water sample 4 times 400 | 800 |
| 12 | 8.3 | Dilute water sample 12 time | 400 | Dilute the water sample 6 times 400 | 800 |
| 16 | 6.2 | Dilute water sample 16 time | 400 | Dilute the water sample 8 times 400 | 800 |
| 24 | 4.2 | Dilute water sample 24 time | 400 | Dilute the water sample 12 times 400 | 800 |
| 32 | 3.1 | Dilute water sample 32 time | 400 | Dilute the water sample 16 times 400 | 800 |

Table S2. LID test results of samples with different retention periods

| Sample type | LID | | | | |
| --- | --- | --- | --- | --- | --- |
|  | 6 h | 48 h | 2 months | St | S6 |
| Us 1 | 3 | 3 | 3 | 0 | 0 |
| Us 2 | 8 | 8 | 6 | 1 | 1.1 |
| Cw 1 | 8 | 8 | 6 | 1 | 1.1 |
| Cw 2 | 4 | 4 | 4 | 0 | 0 |
| Pw 1 | 2 | 3 | 2 | 0.5 | 0.5 |
| Pw 2 | 2 | 2 | 2 | 0 | 0 |
| Ee | 24 | 24 | 24 | 0 | 0 |

*Both 6 h and 48 h were for samples stored under refrigeration at 2℃ to 8℃, and 2 months were for samples stored under refrigeration at -20℃. St is the standard deviation of LID for different sample preservation times, S6 is the standard deviation of LID for 6 independent tests.

Table S3. Influence of different cell densities on the assay method

| Cell density  (cells/ml) | Parallel | Particle density (PCS /ml) | | | Chlorophyll inversion concentration (μg/L) | | | Abs | | |
| --- | --- | --- | --- | --- | --- | --- | --- | --- | --- | --- |
|  |  | Xi | $\bar{X}$ | CV | Xi | $\bar{X}$ | CV | Xi | $\bar{X}$ | CV |
| 3.2×10^3^ | 1 | 3.490×10^3^ | 3.681×10^3^ | 5.14% | 2.40 | 2.43 | 2.50% | 0.004 | 0.004 | 13.32% |
|  | 2 | 3.868×10^3^ |  |  | 2.50 |  |  | 0.004 |  |  |
|  | 3 | 3.684×10^3^ |  |  | 2.39 |  |  | 0.005 |  |  |
| 1.0×10^4^ | 1 | 1.144×10^4^ | 1.146×10^4^ | 0.80% | 7.74 | 7.98 | 3.07% | 0.012 | 0.012 | 8.33% |
|  | 2 | 1.138×10^4^ |  |  | 8.23 |  |  | 0.013 |  |  |
|  | 3 | 1.156×10^4^ |  |  | 7.98 |  |  | 0.011 |  |  |
| 3.2×10^4^ | 1 | 3.530×10^4^ | 3.562×10^4^ | 0.81% | 26.89 | 27.34 | 1.49% | 0.039 | 0.037 | 4.09% |
|  | 2 | 3.570×10^4^ |  |  | 27.46 |  |  | 0.037 |  |  |
|  | 3 | 3.586×10^4^ |  |  | 27.68 |  |  | 0.036 |  |  |
| 1.0×10^5^ | 1 | 1.069×10^5^ | 1.074×10^5^ | 0.65% | 89.14 | 89.35 | 0.70% | 0.116 | 0.113 | 2.71% |
|  | 2 | 1.082×10^5^ |  |  | 90.06 |  |  | 0.11 |  |  |
|  | 3 | 1.071×10^5^ |  |  | 88.86 |  |  | 0.112 |  |  |
| 3.2×10^5^ | 1 | 3.616×10^5^ | 3.627×10^5^ | 1.43% | 282.76 | 279.84 | 0.91% | 0.344 | 0.347 | 0.73% |
|  | 2 | 3.582×10^5^ |  |  | 278.50 |  |  | 0.347 |  |  |
|  | 3 | 3.684×10^5^ |  |  | 278.25 |  |  | 0.349 |  |  |
| 1.0×10^6^ | 1 | 1.061×10^6^ | 1.070×10^6^ | 0.94% | 921.03 | 927.10 | 1.10% | 1.025 | 1.032 | 1.38% |
|  | 2 | 1.069×10^6^ |  |  | 938.87 |  |  | 1.048 |  |  |
|  | 3 | 1.081×10^6^ |  |  | 921.40 |  |  | 1.022 |  |  |

Data are the results of 6 independent tests. Xi is observed value, $\bar{X}$ means average value, CV means coefficient of variation.

Table S4. Interference of sample color on chlorophyll fluorescence analyzer

| Brilliant blue concentration (mg/L) | Parallel | Algal density (cells/mL) | Chlorophyll inversion concentration (μg/L) | | | RSD  (%) | RE  (%) |
| --- | --- | --- | --- | --- | --- | --- | --- |
|  |  |  | Estimated value | After correction | Mean value |  |  |
| 0 | 1 | 1×10^4^ | 9.00 | 7.22 | 7.17 | 1.71% | / |
|  | 2 | 1×10^4^ | 8.81 | 7.03 |  |  |  |
|  | 3 | 1×10^4^ | 9.04 | 7.26 |  |  |  |
|  | 4 | 0 | 1.78 | / | / | / | / |
| 1 | 1 | 1×10^4^ | 15.02 | 7.10 | 6.97 | 2.02% | -2.84% |
|  | 2 | 1×10^4^ | 14.74 | 6.82 |  |  |  |
|  | 3 | 1×10^4^ | 14.90 | 6.98 |  |  |  |
|  | 4 | 0 | 7.92 | / | / | / | / |
| 3.2 | 1 | 1×10^4^ | 25.64 | 6.38 | 6.77 | 7.03% | -5.58% |
|  | 2 | 1×10^4^ | 25.89 | 6.63 |  |  |  |
|  | 3 | 1×10^4^ | 26.56 | 7.30 |  |  |  |
|  | 4 | 0 | 19.26 | / | / | / | / |
| 10 | 1 | 1×10^4^ | 55.12 | 6.26 | 7.18 | 11.33% | 0.09% |
|  | 2 | 1×10^4^ | 56.68 | 7.82 |  |  |  |
|  | 3 | 1×10^4^ | 56.30 | 7.44 |  |  |  |
|  | 4 | 0 | 48.86 | / | / | / | / |
| 32 | 1 | 1×10^4^ | 106.84 | 8.94 | 7.66 | 17.26% | 6.88% |
|  | 2 | 1×10^4^ | 104.20 | 6.30 |  |  |  |
|  | 3 | 1×10^4^ | 105.66 | 7.76 |  |  |  |
|  | 4 | 0 | 97.90 | / | / | / | / |
| 100 | 1 | 1×10^4^ | 162.99 | 6.59 | 6.40 | 18.15% | -10.74% |
|  | 2 | 1×10^4^ | 161.56 | 5.16 |  |  |  |
|  | 3 | 1×10^4^ | 163.86 | 7.46 |  |  |  |
|  | 4 | 0 | 156.40 | / | / | / | / |

Table S5. Interference of sample color to spectrophotometer determination

| Brilliant blue concentration (mg/L) | Parallel | Algal density  (cells/ml) | Abs | | | RSD  (%) | RE  (%) |
| --- | --- | --- | --- | --- | --- | --- | --- |
|  |  |  | Estimated value | After correction | Mean value |  |  |
| 0 | 1 | 1×10^4^ | 0.012 | 0.012 | 0.012 | 4.68% | / |
|  | 2 | 1×10^4^ | 0.012 | 0.012 |  |  |  |
|  | 3 | 1×10^4^ | 0.013 | 0.013 |  |  |  |
|  | 4 | 0 | 0 | / | / | / | / |
| 1 | 1 | 1×10^4^ | 0.016 | 0.011 | 0.012 | 4.95% | -5.41% |
|  | 2 | 1×10^4^ | 0.017 | 0.012 |  |  |  |
|  | 3 | 1×10^4^ | 0.016 | 0.011 |  |  |  |
|  | 4 | 0 | 0.005 | / | / | / | / |
| 3.2 | 1 | 1×10^4^ | 0.031 | 0.014 | 0.013 | 4.33% | 8.11% |
|  | 2 | 1×10^4^ | 0.031 | 0.014 |  |  |  |
|  | 3 | 1×10^4^ | 0.030 | 0.013 |  |  |  |
|  | 4 | 0 | 0.017 | / | / | / | / |
| 10 | 1 | 1×10^4^ | 0.084 | 0.016 | 0.015** | 3.77% | 24.32% |
|  | 2 | 1×10^4^ | 0.084 | 0.016 |  |  |  |
|  | 3 | 1×10^4^ | 0.083 | 0.015 |  |  |  |
|  | 4 | 0 | 0.068 | / | / | / | / |
| 32 | 1 | 1×10^4^ | 0.271 | 0.025 | 0.022** | 10.50% | 78.38% |
|  | 2 | 1×10^4^ | 0.267 | 0.021 |  |  |  |
|  | 3 | 1×10^4^ | 0.267 | 0.021 |  |  |  |
|  | 4 | 0 | 0.246 | / | / | / | / |
| 100 | 1 | 1×10^4^ | 1.051 | 0.065 | 0.061** | 20.59% | 391.89% |
|  | 2 | 1×10^4^ | 1.033 | 0.047 |  |  |  |
|  | 3 | 1×10^4^ | 1.057 | 0.071 |  |  |  |
|  | 4 | 0 | 0.986 | / | / | / | / |

Significant differences were indicated with **p* < 0.05, ***p* < 0.01, and ****p* < 0.001.

Table S6. Interference of particulate matter in the determination of chlorophyll fluorescence

| Porcelain powder concentration (NTU) | Parallel | Algal density  (cells/mL) | Particle concentration  (counts/mL) | | | RSD  (%) | RE  (%) |
| --- | --- | --- | --- | --- | --- | --- | --- |
|  |  |  | Estimated value | After correction | Mean value |  |  |
| 0 | 1 | 1×10^4^ | 9.00 | 7.22 | 7.17 | 1.71% | / |
|  | 2 | 1×10^4^ | 8.81 | 7.03 |  |  |  |
|  | 3 | 1×10^4^ | 9.04 | 7.26 |  |  |  |
|  | 4 | 0 | 1.78 | / | / | / | / |
| 25 | 1 | 1×10^4^ | 9.02 | 6.96 | 7.37 | 4.90% | 2.79% |
|  | 2 | 1×10^4^ | 9.59 | 7.53 |  |  |  |
|  | 3 | 1×10^4^ | 9.69 | 7.63 |  |  |  |
|  | 4 | 0 | 2.06 | / | / | / | / |
| 50 | 1 | 1×10^4^ | 10.06 | 7.55 | 7.87 | 7.80% | 9.72% |
|  | 2 | 1×10^4^ | 11.08 | 8.57 |  |  |  |
|  | 3 | 1×10^4^ | 9.98 | 7.47 |  |  |  |
|  | 4 | 0 | 2.51 | / | / | / | / |
| 100 | 1 | 1×10^4^ | 11.10 | 7.40 | 7.04 | 4.80% | -1.86% |
|  | 2 | 1×10^4^ | 10.69 | 6.99 |  |  |  |
|  | 3 | 1×10^4^ | 10.43 | 6.73 |  |  |  |
|  | 4 | 0 | 3.70 | / | / | / | / |
| 200 | 1 | 1×10^4^ | 12.69 | 6.48 | 7.11 | 7.76% | -0.84% |
|  | 2 | 1×10^4^ | 13.73 | 7.52 |  |  |  |
|  | 3 | 1×10^4^ | 13.53 | 7.32 |  |  |  |
|  | 4 | 0 | 6.21 | / | / | / | / |
| 400 | 1 | 1×10^4^ | 16.37 | 7.02 | 7.51 | 6.21% | 4.70% |
|  | 2 | 1×10^4^ | 17.30 | 7.95 |  |  |  |
|  | 3 | 1×10^4^ | 16.90 | 7.55 |  |  |  |
|  | 4 | 0 | 9.35 | / | / | / | / |

Table S7. Interference of sample particulate matter to electronic particle counter measurement

| Porcelain powder concentration(NTU) | Parallel | Algal density  (cells/mL) | Particle concentration (counts/mL) | | | RSD  (%) | RE  (%) |
| --- | --- | --- | --- | --- | --- | --- | --- |
|  |  |  | Estimated value | After correction | Mean value |  |  |
| 0 | 1 | 1×10^4^ | 1.129×10^4^ | 1.057×10^4^ | 1.052×10^4^ | 1.77% | / |
|  | 2 | 1×10^4^ | 1.139×10^4^ | 1.067×10^4^ |  |  |  |
|  | 3 | 1×10^4^ | 1.103×10^4^ | 1.031×10^4^ |  |  |  |
|  | 4 | 0 | 7.160×10^2^ | / | / | / | / |
| 25 | 1 | 1×10^4^ | 1.418×10^4^ | 1.038×10^4^ | 1.067×10^4^ | 4.23% | 1.39% |
|  | 2 | 1×10^4^ | 1.499×10^4^ | 1.119×10^4^ |  |  |  |
|  | 3 | 1×10^4^ | 1.424×10^4^ | 1.044×10^4^ |  |  |  |
|  | 4 | 0 | 3.803×10^3^ | / | / | / | / |
| 50 | 1 | 1×10^4^ | 2.024×10^4^ | 1.051×10^4^ | 1.065×10^4^ | 1.67% | 1.19% |
|  | 2 | 1×10^4^ | 2.032×10^4^ | 1.059×10^4^ |  |  |  |
|  | 3 | 1×10^4^ | 2.058×10^4^ | 1.085×10^4^ |  |  |  |
|  | 4 | 0 | 9.734×10^3^ | / | / | / | / |
| 100 | 1 | 1×10^4^ | 3.810×10^4^ | 1.167×10^4^ | 1.132×10^4^ | 2.77% | 7.63% |
|  | 2 | 1×10^4^ | 3.749×10^4^ | 1.106×10^4^ |  |  |  |
|  | 3 | 1×10^4^ | 3.767×10^4^ | 1.124×10^4^ |  |  |  |
|  | 4 | 0 | 2.643×10^4^ | / | / | / | / |
| 200 | 1 | 1×10^4^ | 9.234×10^4^ | 6.690×10^3^ | 9.000×10^3^ | 22.90% | -14.45% |
|  | 2 | 1×10^4^ | 9.531×10^4^ | 9.660×10^3^ |  |  |  |
|  | 3 | 1×10^4^ | 9.630×10^4^ | 1.065×10^4^ |  |  |  |
|  | 4 | 0 | 8.565×10^4^ | / | / | / | / |
| 400 | 1 | 1×10^4^ | 2.817×10^5^ | 1.242×10^4^ | 6.960×10^3^ | 85.13% | -33.84% |
|  | 2 | 1×10^4^ | 2.771×10^5^ | 7.800×10^3^ |  |  |  |
|  | 3 | 1×10^4^ | 2.699×10^5^ | 6.600×10^2^ |  |  |  |
|  | 4 | 0 | 2.693×10^5^ | / | / | / | / |

Table S8. Interference of sample particulate matter to spectrophotometer determination

| Porcelain powder concentration (NTU) | Parallel | Algal density  (cells/mL) | Particle concentration (counts/mL) | | | RSD  (%) | RE  (%) |
| --- | --- | --- | --- | --- | --- | --- | --- |
|  |  |  | Estimated value | After correction | Mean value |  |  |
| 0 | 1 | 1×10^4^ | 0.012 | 0.012 | 0.012 | 4.68% | / |
|  | 2 | 1×10^4^ | 0.012 | 0.012 |  |  |  |
|  | 3 | 1×10^4^ | 0.013 | 0.013 |  |  |  |
|  | 4 | 0 | 0 | / | / | / | / |
| 25 | 1 | 1×10^4^ | 0.193 | 0.006 | 0.004 | 144.31% | -70.27% |
|  | 2 | 1×10^4^ | 0.185 | -0.002 |  |  |  |
|  | 3 | 1×10^4^ | 0.195 | 0.008 |  |  |  |
|  | 4 | 0 | 0.187 | / | / | / | / |
| 50 | 1 | 1×10^4^ | 0.341 | 0.018 | 0.020 | 10.24% | 64.86% |
|  | 2 | 1×10^4^ | 0.344 | 0.021 |  |  |  |
|  | 3 | 1×10^4^ | 0.345 | 0.022 |  |  |  |
|  | 4 | 0 | 0.323 | / | / | / | / |
| 100 | 1 | 1×10^4^ | 0.639 | 0.048 | 0.042 | 23.18% | 243.24% |
|  | 2 | 1×10^4^ | 0.639 | 0.048 |  |  |  |
|  | 3 | 1×10^4^ | 0.622 | 0.031 |  |  |  |
|  | 4 | 0 | 0.591 | / | / | / | / |
| 200 | 1 | 1×10^4^ | 1.125 | 0.018 | 0.021 | 10.07% | 67.57% |
|  | 2 | 1×10^4^ | 1.129 | 0.022 |  |  |  |
|  | 3 | 1×10^4^ | 1.128 | 0.021 |  |  |  |
|  | 4 | 0 | 1.107 | / | / | / | / |
| 400 | 1 | 1×10^4^ | 1.980 | 0.047 | 0.047 | 7.42% | 283.78% |
|  | 2 | 1×10^4^ | 1.984 | 0.051 |  |  |  |
|  | 3 | 1×10^4^ | 1.977 | 0.044 |  |  |  |
|  | 4 | 0 | 1.933 | / | / | / | / |

Table S9. Results of algal growth inhibition test on reference substance

| Sample type | Test number ^a^ | Concentration (mg/L) | Suppression ratio | | | | Average EC_50_ | 95% confidence interval |
| --- | --- | --- | --- | --- | --- | --- | --- | --- |
|  |  |  | $\bar{X}$ | Si | | CV |  |  |
| 3, 5-dichlorophenol | 6 | 4.00 | 107.2% | | 5.0% | 4.7% | 3.20 | 3.15-3.26 |
|  |  | 3.48 | 70.5% | | 3.0% | 4.3% |  |  |
|  |  | 3.02 | 34.8% | | 3.1% | 8.8% |  |  |
|  |  | 2.63 | 21.1% | | 3.9% | 18.4% |  |  |
|  |  | 2.29 | 12.3% | | 3.6% | 29.3% |  |  |
|  |  | 1.99 | 3.6% | | 3.1% | 86.0% |  |  |
|  |  | 1.73 | 0.2% | | 1.3% | 717.1% |  |  |
|  |  |  |  | |  |  |  |  |
| potassium dichromate | 6 | 1.44 | 71.3% | | 1.9% | 2.6% | 1.10 | 1.08-1.13 |
|  |  | 1.20 | 58.4% | | 3.4% | 5.8% |  |  |
|  |  | 1.00 | 40.1% | | 1.7% | 4.2% |  |  |
|  |  | 0.83 | 23.0% | | 4.4% | 19.3% |  |  |
|  |  | 0.69 | 13.3% | | 3.4% | 25.4% |  |  |
|  |  | 0.58 | 4.8% | | 2.6% | 54.5% |  |  |

^a^ Data are the results of 6 independent tests. $\bar{X}$ means average value, Si means standard deviation, CV means coefficient of variation.


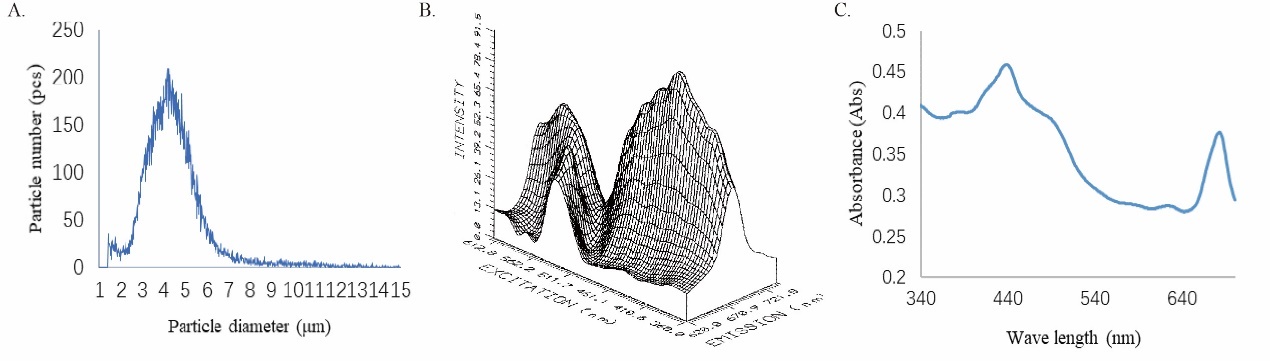


Figure S1. Self-characterization of *R. subcapitata*. A) Particle size distribution of *R. subcapitata*. The abscissa represents the particle diameter and the ordinate represents the particle number. B) Fluorescence map of *R.* *subcapitata*. C) Absorption spectrum of *R. subcapitata*. The abscissa represents the wavelength and the ordinate represents the absorbance.


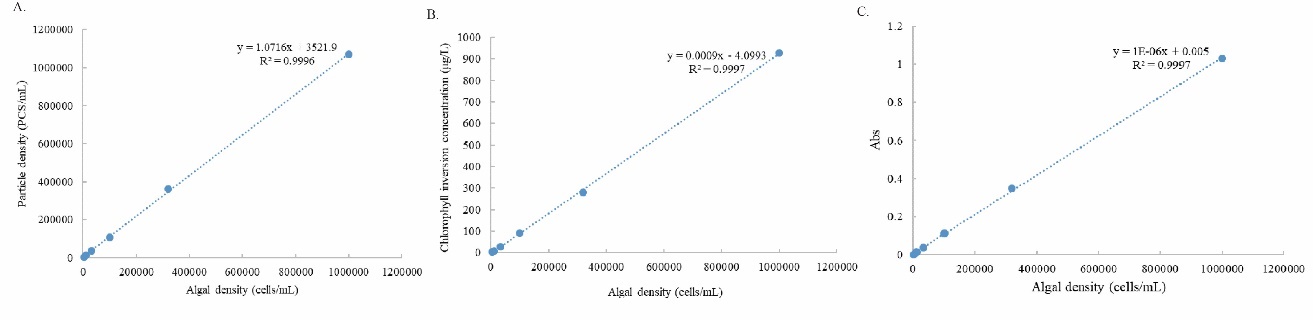


Figure S2. Correlation between different biomass measurement methods and algal density. The abscissa is the algae density. A) The ordinate is the particle density. B) The ordinate is the chlorophyll inversion concentration. C) The ordinate is the absorbance.
